# Rifampicin-Induced Panic Disorder in a 70-Year-Old Woman With Brucellosis: A Case Report

**DOI:** 10.1192/bjo.2025.10701

**Published:** 2025-06-20

**Authors:** Sunday Adeoye

**Affiliations:** Humber Teaching NHS Foundation Trust, Hull, United Kingdom

## Abstract

**Aims:** Panic disorder, also known as paroxysmal episodic anxiety, is a severe, unpredictable, and debilitating form of anxiety that is characterised by sudden onset of palpitations, chest pain, choking sensations, dizziness, and feelings of unreality (depersonalization or derealization). People that suffer from panic attack often have a secondary fear of dying or losing control. Like many other mental illnesses, the causes of panic disorder are unknown, but some predisposing factors have been identified including genetics, neurobiology, medications, psychiatric disorders, psychosocial factors and medical conditions.

Brucellosis is an infective medical condition caused by Brucella. In the UK, it is a very rare notifiable disease and transmission is usually from consumption of unpasteurised milk and cheese or from contact with infected animals. Brucellosis is considered a public health problem by many countries not only because of the economic loss it brings but it also causes chronic pain, anxiety, and depression, affecting the life quality of patients. Treatment for human brucellosis is based on combinations of antibiotics such as doxycycline, streptomycin, gentamicin, ciprofloxacin, and trimethoprim–sulfamethoxazole and rifampicin over a period of about 6 weeks. Rifampicin on the other hand has been implicated in worsening mental health conditions like anxiety and depression in many previous literatures.

The mechanism by which this occurs has been put to the drug-to-drug interaction that rifampicin might have with other medication patients are taking and the effect of rifampicin as cytochrome p450 enzyme inducers. This case report presents another perspective of rifampicin inducing and worsening panic disorder in a patient that has not previously been diagnosed with anxiety disorder.

**Methods:** This patient was a 70-year-old woman with background of depression, stable on sertraline, who contracted canine brucellosis from her dog. While on isolation ward in physical health hospital, she was commenced on rifampicin intravenously and oral doxycycline. She developed new onset panic attack five days into the treatment. was referred by the General practitioner following reports of new onset panic attacks. She continued to suffer from these panic attacks even when rifampicin was changed to oral after discharge from hospital. This affected her quality of life significantly. She was commenced on short course benzodiazepine after risk and benefit explained to her. Her sertraline was increased from 50 mg to 100 mg daily. Other psychological options were also explored. Patient reported good improvement within two weeks of commencement of treatment.

She reported good improvement during four-week follow up visit. She had not had panic attacks two weeks prior to the review. She had been tapered off the diazepam and she was one week left in completing her antibiotic regimen. Patient was followed up for two more weeks and discharged back to the GP.

**Results:** This patient presented with new onset panic attacks following commencement on rifampicin in the treatment of brucellosis. There have been reports that anxiety and depression are the commonest mental health conditions associated with brucellosis. It is therefore a possibility that the new severe debilitating form of anxiety is due to the infection with Brucella. On the other hand, there have been reports that patients treated for tuberculosis with rifampicin have increased incidence of anxiety. The mechanism of this has been linked to the inducing effect rifampicin has on liver enzyme cytochrome P450 which plays a crucial role in the metabolism of most medications. In the case of index patient, rifampicin might have had a drug-drug interaction with sertraline this patient was taking for depression. Though the sertraline was intended for treatment of depression, it is obvious that it was also helpful for premorbid anxiety for this patient. Once the beneficial effect of sertraline was reduced by the rifampicin, it led to the onset of severe and debilitating anxiety. Similar reports about interaction between rifampicin and citalopram have been reported.

**Conclusion:** The follow-up to this report would be to conduct a case series and observe the occurrence of drug-drug interaction between rifampicin and other common psychotropics.

